# Two Rare Cases of Uterine Leiomyosarcomas Originating from Submucosal Leiomyomas Proved by Their Immunohistochemistry Profiles

**DOI:** 10.22074/ijfs.2020.6222

**Published:** 2020-10-12

**Authors:** Hossein Ghorbani, Mohammad Ranaee, Zeinab Vosough

**Affiliations:** Department of Pathology, Babol University of Medical Sciences, Babol, Iran

**Keywords:** Immunohistochemistry, Leiomyoma, Leiomyosarcoma, Uterus

## Abstract

The most common mesenchymal tumours of the uterine corpus originate from smooth muscle cells. Leiomyomas are commonly found in women of child bearing age; however, leiomyosarcomas occur later in life (50-55 years of age). Most uterine leiomyosarcomas occur de novo, but rare cases of leiomyosarcomas that arise from leiomyomas have been reported. We present two cases of fertile women with submucosal leiomyomas that became malignant and dis- cuss their pathologic features and immunohistochemistry studies for P16, P53 and Ki67.

## Introduction

The most common mesenchymal tumours of the uterus are the smooth muscle tumours (1). The majority of these tumours are leiomyomas. Leiomyosarcomas occur rarely, around 1 in 800 smooth muscle tumour cases (2). Some of these tumours present with diagnostic challenges. The most frequently used parameters to differentiate these tumours are the extent and degree of atypia, coagulative necrosis and mitotic activity of the tumour (1). Tumour cellularity, vascular and myometrial invasion, tumour cell differentiation and presence of giant cells are beneficial. Leiomyomas are very common and present in 20-30% of women aging over 30 years; however, leiomyosarcomas account for only 1.3% of uterine malignancies. Most uterine leiomyosarcomas occur de novo but rare cases of leiomyosarcomas that arise from leiomyomas have been reported (3). In this paper, we report two cases of this rare phenomenon. Both patients gave consent for using their clinical data in research.

### Case 1

**Fig.1:**
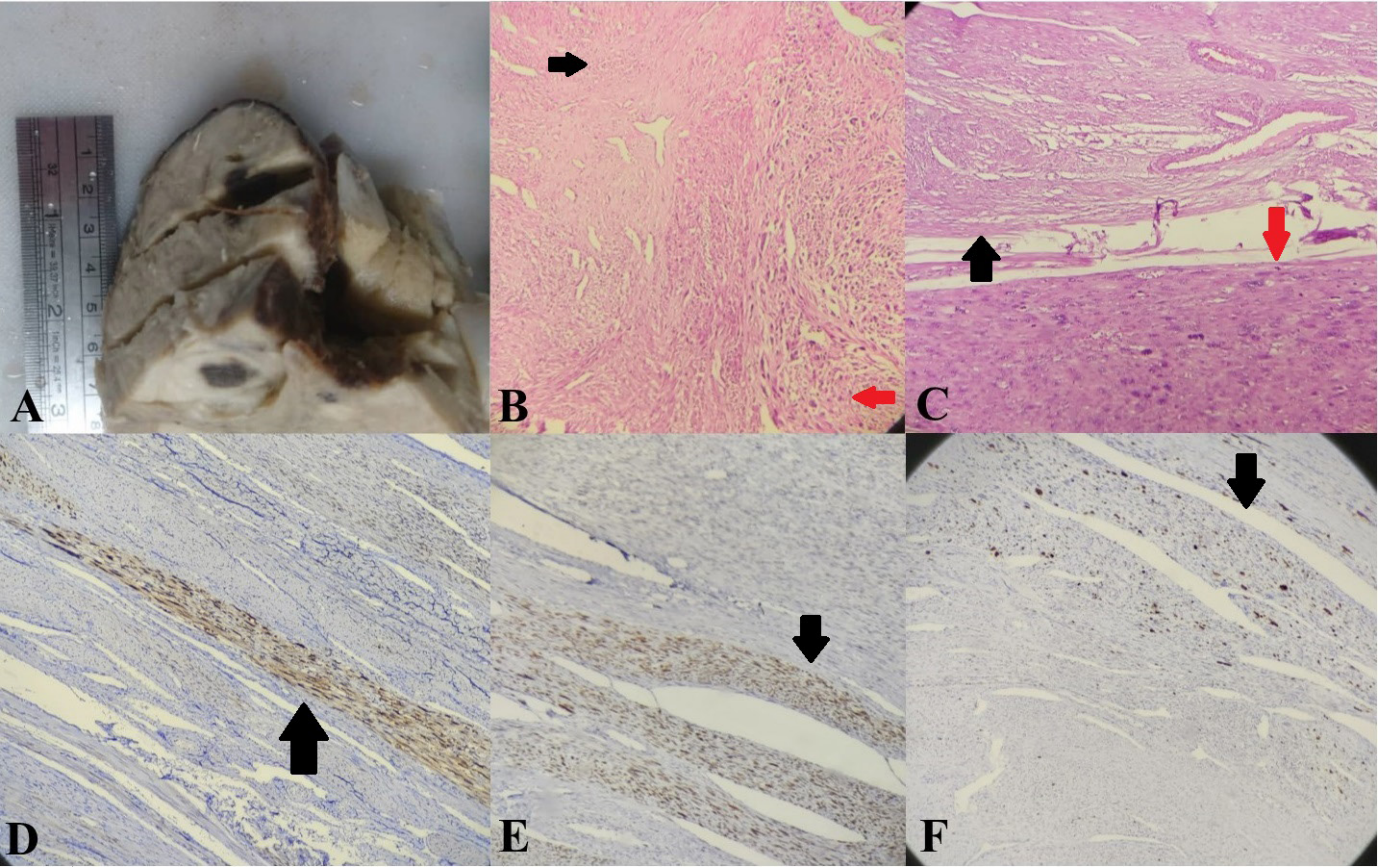
Case 1. **A.** Gross appearance of the tumour. **B. **Leiomyomatous area on the left (black arrow) opposed to leiomyosarcomatous area on the right (red arrow). Haematoxylin and eosin (H&E) staining, x40. **C.** Leiomyomatous area on top opposed to leiomyosarcomatous area on the bottom (H&E staining, 100x). **D.** P16, **E.** P53, and **F. **Ki67 were all positive in the leiomyosarcomatous areas (arrows).

A 41-year-old woman (gestation 3, labour 3) presented to the gynaecology clinic with menometrorrhagia. Her past medical history and physical examination, including vaginal exam, were normal. On transabdominal ultrasound, the uterine size was 117x68 mm with a homogenous myometrial echo. A solid mass that measured 63x43x52 mm with mixed echogenicity filled the endometrial cavity and was suggestive of a submucosal leiomyoma. The adnexa were normal. No other abnormal abdominopelvic findings were identified. The patient underwent a total abdominal hysterectomy and bilateral salpingo-oophorectomy. During the surgery, an omental adhesion was identified, which the surgeon decided to send for abdominal cytology. On gross examination of the specimen in the uterus, a 7 cm diameter submucosal mass and three (1.5, 0.8 and 0.3 cm diameter) intramural masses were found. The largest mass had a creamy cut surface with areas of haemorrhage and the smaller masses had homogenous creamy cut surfaces (Fig . 1A). A cyst filled with clear watery fluid was identified in the right ovary. Histologic examination of the submucosal mass showed a classic leiomyoma appearance except for multiple foci of nuclear atypical features and a high mitotic index (Fig. 1B, C). The intramural masses were diagnosed as leiomyomas. The cytology was negative. Immunohistochemistry revealed P16 and P53 nuclear staining and a high Ki67 index in leiomyosarcomatous areas, but not in any other areas (Fig . 1D-F).

### Case 2

**Fig.2:**
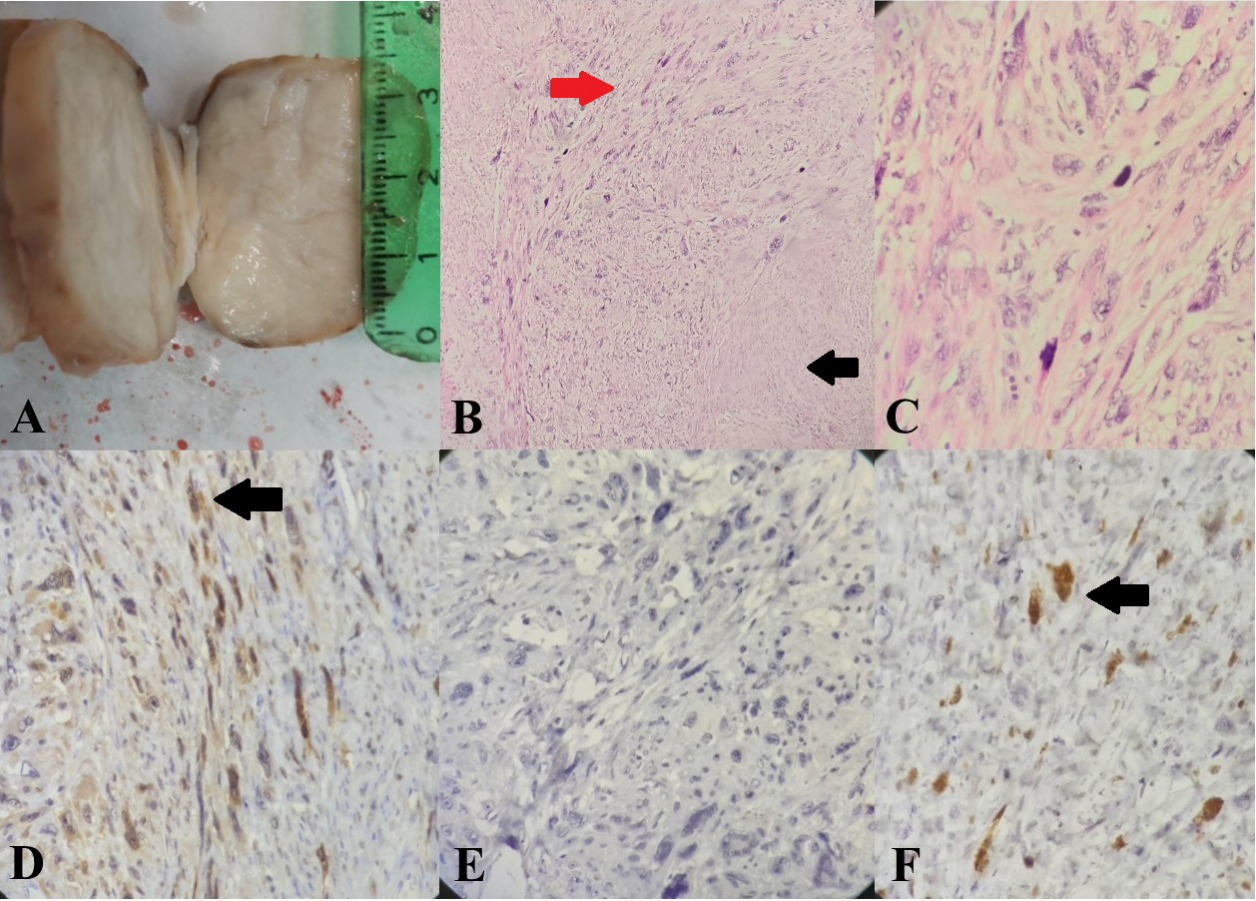
Case 2. **A.** Gross appearance of the tumour.** B.** Leiomyomatous area on the right (black arrow) opposed to leiomyosarcomatous area on the left (red arrow). Haematoxylin and eosin (H&E) staining, x40. **C.** Leiomyosarcomatous area (H&E staining, x400). **D.** P16, **E.** P53, and **F. **Ki67 (Positive staining are identified by arrows).

A 35-year-old woman (gestation 2, labour 1, abortion 1) went to the Gynaecology Clinic with abnormal uterine bleeding and spotting. Her drug history revealed consumption of a vaginal herbal suppository and oral contraceptive during the last year. Vaginal examination revealed a mass lesion that projected from the cervical canal. Transvaginal ultrasound showed a uterine that was 102x55x31 mm in size, endometrial thickness of 4 mm and a submucosal mass in the cervical canal that measured 54x46 mm. No abnormality was identified in the adnexa. The patient underwent a transvaginal myomectomy surgery. The surgical specimen was a round, polypoid creamy mass that measured 5.5x4x2.5 cm. The cut surface was homogenous and creamy with a typical whorled pattern (Fig . 2A). Microscopic sections revealed conventional leiomyoma with some foci of increased cellularity, nuclear pleomorphism and atypia with numerous mitotic figures with some atypical ones (Figure 2B, C). Immuno histochemistry was positive for P16 nuclear staining and a high Ki67 index in these areas, but P53 was negative (Fig . 2D-F)

## Discussion

Two types of acceptable theories for the origin of leyomyosarcomas are presented in the literature: a) uterine leiomyomas have no malignancy potential and leiomysarcomas are a de novo process without pre-existing neoplastic lesions (1) and b) rarely, leiomyomas can undergo malignant changes and become leiomyosarcomas (4).

We report two cases of uterine leiomyosarcomas that came from leiomyomas. The mean age of leiomyosarcoma presentation is 10 years more than leiomyomas (50-55 years) (1) and the most frequent symptoms are abnormal vaginal bleeding, abdominal pain and presence of an intramural mass. Concerning age, both women in our cases were younger than expected, but the initial symptom was abnormal vaginal bleeding in both. Solitary lesions occur more frequently in leiomyosarcomas than in their benign counterparts (3). Like other previously reported cases, one of our cases had multiple leiomyomas (5, 6). Leiomyosarcomas are most commonly intramural masses that measure around 6-9 cm in diameter (1); our cases were localized in the submucosa and were smaller in size compared to most de novo leiomyosarcomas.

The parallel between the current case features and leiomyosarcomas shows the unpredictable appearance that a leiomyoma undergoing malignant changes can take. Fortunately, the limited number of reported cases (with the exception of a few) (6-8), had no recurrences or metastases during long-term follow up (5, 9-11).

The accepted criteria for leiomyosarcoma consists of high mitotic figures, nuclear atypia and coagulative necrosis. In our cases, the necrosis was absent but the presence of the two other features differentiated them from smooth muscle tumours of uncertain malignant potential (STUMP) with regards to the current diagnostic criteria (1). Under these conditions, an appropriate good question is: ‘When the tumour does not have all the characteristic features of a leiomyosarcoma, what are the alternative tools to help with diagnosis?’ We ordered immunohistochemistry studies for some of the available markers that were used in previous studies.

In 2009, Mittal et al. (12) performed an immunohistochemistry examination of 26 leiomyosarcomas that had benign looking areas. They scored the stainings of P53, oestrogen receptor (ER), progesterone receptor (PR) and Ki67 index in both the leiomyosarcoma and leiomyoma areas. They attempted to detect genetic aberrations by means of high density oligonucleotide array (CGH array). The results showed that ER and PR were lower in leiomyosarcomas areas compared to leiomyomas, but Ki67 index and P53 scores were higher in these tumours. In addition leiomyoma-like areas presented with alterations of numerous oncogenes, transcription factors and tumour suppressor genes. The proposed theory was that not all, but only rare cases, of leiomyomas could be precancerous lesions.

The investigation of p53 mutation in uterine smooth muscle tumours was first conducted by De Vos et al. They sequenced the P53 exons of eight cases of leiomyomas and eight cases of leiomyosarcomas. Point mutations were observed in three cases from the leiomyosarcoma group, whereas none of the leiomyomas showed any alterations (13). Subsequently, many studies used p53 immunohistochemistry as a helpful diagnostic tool; however, it was uncommon to use p16 (6, 10, 11). We performed immunohistochemistry for the P53 and P16 markers, and the Ki67 index for both cases. The results showed positive P16 staining and high Ki67 index coloration in both cases. P53 staining was observed in only one case. The pattern of staining showed that, other than the positive areas, the remaining sections were similar to classic leiomyoma. Therefore, we concluded that the malignancy arose from a benign tumour. In cases with similar features, immunohistochemistry for both P53 and P16 could be a useful tool in proving the malignant nature of these areas. Utilization of both markers was quite unique and there have been few studies. Thus, we recommend further investigations of these cases.

## Conclusion

This study used immunohistochemistry for the P53, P16 markers and the Ki67-index to confirm leiomyosarcomas that arose from small submucosal leiomyomas in two young women. These findings raise the possibility of malignant transformation of very benign looking leiomyomas.
